# Pertussis seroepidemiology in women and their infants in Sarlahi District, Nepal

**DOI:** 10.1016/j.vaccine.2017.09.074

**Published:** 2017-12-04

**Authors:** Michelle M. Hughes, Janet A. Englund, Kathryn Edwards, Sandra Yoder, James M. Tielsch, Mark Steinhoff, Subarna K. Khatry, Steven C. LeClerq, Joanne Katz

**Affiliations:** a Johns Hopkins Bloomberg School of Public Health, Department of International Health, Global Disease Epidemiology and Control, 615 North Wolfe Street, Baltimore, MD 21205, USA; b University of Washington, Seattle Children’s Hospital, 4800 Sand Point Way NE MA 7.234, Seattle, WA 98105, USA; c Vanderbilt University, Vanderbilt Vaccine Research Program, 21st Avenue South, Nashville, TN 37232, USA; d George Washington University Milken Institute School of Public Health, Department of Global Health, 950 New Hampshire Avenue, WA, DC 20052, USA; e Cincinnati Children’s Hospital and Medical Center, Global Health Center, R8508, 3333 Burnet Avenue, Cincinnatti, OH 45229, USA; f Nepal Nutrition Intervention Project–Sarlahi, Kathmandu, Nepal

**Keywords:** Pertussis, Maternal antibodies, Immunoglobulin G transfer, Nepal

## Abstract

**Background:**

Infants are at greatest risk for pertussis morbidity and mortality. Maternal vaccination during pregnancy has been shown to prevent pertussis in young infants in high- and middle-income countries. However, data on the levels of maternal pertussis antibodies and the efficiency of transplacental transfer in low-income South Asian settings are limited.

**Objective:**

To estimate the prevalence of maternal pertussis antibodies and the efficiency of transplacental transfer in rural southern Nepal.

**Design/methods:**

Paired maternal-infant blood samples were collected from a subsample of participants in a randomized, controlled trial of maternal influenza immunization (n = 291 pairs). Sera were tested by enzyme-linked immunosorbent assays for pertussis toxin, filamentous hemagglutinin, pertactin, and fimbriae. Maternal and infant pertussis antibody levels and transplacental transfer efficiency were determined and potential factors associated with both were assessed.

**Results:**

Elevated maternal antibodies to pertussis toxin, suggesting recent pertussis infection, were rarely detected (4%, tested n = 305). However, paired maternal-cord sera were highly correlated across all antibodies; transplacental antibody transfer ratios for pertussis toxin were 1.14 (n = 291, 95% CI 1.07–1.20); filamentous hemagglutinin 1.10 (n = 120, 95% CI: 1.01–1.20); fimbriae 2/3 1.05 (n = 120, 95% CI: 0.96–1.15) and pertactin 0.96 (n = 289, 95% CI: 0.91–1.00). Older gestational age was associated with increased pertussis toxin and decreased fimbriae 2/3 antibody transport.

**Conclusions:**

A low prevalence of maternal antibody to all four pertussis antigens was noted in Nepal, but transplacental antibody transfer was efficient. No consistent demographic factors were associated with elevated maternal antibody levels or efficiency of transplacental transfer. If an increase in infant pertussis disease burden was detected in this population, maternal immunization could be an effective intervention to prevent disease in early infancy.

## 1 Introduction

Epidemic levels of pertussis have been reported recently, mainly in high-income countries where acellular vaccines are exclusively used [1], [2], [3]. Age groups particularly affected include infants and adolescents [4], [5]. The resurgence of infant pertussis is of greatest concern as infants are at highest risk for severe morbidity and mortality, particularly before they begin their primary pertussis vaccination series [1], [6].

Although adolescent and adult boosters [7] and “cocooning” of infant caregivers by vaccination have been attempted, these approaches have not effectively decreased infant pertussis burden [1], [8]. The most promising strategy that has recently been implemented in several high- and middle-income countries has been the vaccination of women during pregnancy [9], [10], [11]. This approach boosts maternal antibodies, providing protection to both mothers and infants, through transplacental antibody transport and potential augmentation of breastmilk antibody [12], [13], [14].

While several studies have examined the level of maternal and cord blood pertussis antibodies and the efficiency of transplacental antibody transfer [15], [16], [17], [18], [19], [20], [21], [22], [23], [24], [25], [26], [27], few studies have been conducted in low-income country settings where whole cell diphtheria-tetanus-pertussis vaccine is exclusively used in infants and rates of maternal and infant malnutrition and prematurity are high [14]. Thus, we sought to determine maternal and cord blood antibody levels to four pertussis antigens, the efficiency of transplacental antibody transfer, and those factors that modify these levels.

## 2 Methods

### 2.1 Settings and population

The study was nested within a randomized controlled trial of maternal influenza vaccination during pregnancy conducted within a population of about 98,000 individuals in 9 administrative Village Development Communities in Sarlahi District, Nepal [28], [29], [30]. In brief, pregnancies were identified through a pregnancy surveillance system (women 15–40 years) where field workers visited all households at 5 week intervals and performed urine pregnancy tests. Between April 25, 2011 and September 9, 2013, 3,693 women, between 17 and 34 weeks gestation, who consented to participate, were enrolled and randomized to receive either influenza vaccine or placebo in two consecutive cohorts. A substudy of pertussis antibody prevalence was conducted in a convenience sample of women and their liveborn infants from whom blood samples were collected, starting approximately 10 months after the main trial began. Study approval was obtained from Institutional Review Boards at the Johns Hopkins Bloomberg School of Public Health, Cincinnati Children’s Medical Center, Institute of Medicine at Tribhuvan University, Kathmandu, and the Nepal Health Research Council. The trial is registered at Clinicaltrials.gov (NCT01034254).

### 2.2 Data collection

Baseline demographic and household data were collected at enrollment. Household size was dichotomized at the median (≤4 versus > 4 people). Responses to twenty-five questions were used to develop a household socioeconomic (SES) construct [31]. Results were averaged and divided into SES quartiles for analysis. Once a pregnancy was identified, women reported their literacy status (binary), number of pregnancies, and date of last menstrual period. For parity analysis, women were categorized as nulliparous or parous. Field workers identified maternal ethnicity (Pahadi or Madeshi).

Mothers were requested to collect between 2 and 5 cubic centimeters of umbilical cord blood either in the home or at the local delivery center as soon as the placenta was delivered. The woman or her representative contacted the local study team and they collected birth information, retrieved the cord blood samples, and measured infant weight. For mothers who delivered in a health facility, cord blood was collected by facility staff, who notified the study team. Maternal blood was collected approximately 1 week post-partum in the home setting. All blood was transported on ice to the central field-processing laboratory for centrifugation. Sera were removed, aliquoted into cryovials and stored in liquid nitrogen.

Gestational age was determined using date of last menstrual period recorded during pregnancy surveillance. Those infants who delivered at <37 weeks were categorized as preterm, but were not excluded from the antibody study. Birthweight was obtained at home after birth using a digital scale; those children with weights collected >72 hours after birth were excluded from the analysis. Infants were categorized as low birthweight if <2500 g. Small for gestational age (SGA) was calculated using INTERGROWTH-21 sex-specific 10th percentile cut-off standards [32].

### 2.3 Laboratory assays

Immunoglobulin G (IgG) anti-pertussis toxin (PT), pertactin (PRN), filamentous hemagglutinin (FHA), and fimbriae 2/3 (FIM) enzyme-linked immunosorbent assays (ELISA) were performed at Vanderbilt University School of Medicine according to previously described methods [33]. ELISA units were assigned based on the US Food and Drug Administration human reference pertussis antisera lot 3 for PT and FHA and lot 4 for PRN. An internal Vanderbilt standard was used to calibrate FIM. The lower level of quantification (LOQ) for each antigen was 10 ELISA units (EU)/mL.

### 2.4 Analytic dataset

Mother-infant pairs were included in the initial testing list if both delivery cord and maternal post-partum blood samples were collected (Supplemental Fig. 1). Samples with inadequate quantity of sera for analysis were excluded; this resulted in some women not having paired infant sera due to insufficient infant sera volume. Due to sample availability and funding constraints, PT and PRN testing were prioritized, resulting in different sample sizes for the different pertussis antibodies. More details on the full sample selection process may be found in Supplemental Fig. 1.

### 2.5 Statistical analysis

Maternal and infant antibody levels below the LOQ were assigned one-half of the assay LOQ (5 EU/mL) and geometric mean concentrations (GMC) and the percent below the LOQ were determined. Reverse cumulative distribution curves compared the distribution of log transformed antibody titers [34]. Differences in maternal pertussis antibody levels by maternal, infant, and household characteristics were compared using non-parametric testing for binary (Wilcoxon rank sum test with continuity correction) and nominal (Kruskal-Wallis rank sum test) variables. Maternal antibody titers that met or exceeded 94 EU/mL for PT were defined as recently infected [35]; PT is the only antigen seen with pertussis infection and none of the mothers had been recently immunized with pertussis containing vaccines.

The ratio of infant to maternal pertussis antibody GMC was calculated and correlated using the Spearman’s rank correlation. A multivariable linear regression model was developed to examine the association of the log ratio of infant to maternal pertussis antibody levels with infant, maternal, and household characteristics. We did not use a specific p-value cut-off for inclusion and excluded some covariates that were closely associated in the final model (for example, low birth weight and SGA).

The cutoff for statistical significance in all testing was p < .05. Statistical analyses were conducted in R version 3.3.2 (2016-10-31) and Stata 14.2.

## 3 Results

305 mother and 291 infant blood samples collected between March 1, 2012 and October 30, 2013 were analyzed (Supplemental Fig. 1). The 305 maternal blood samples were collected from 4-38 days post-partum (median = 10 days). Average maternal age was 24 years compared to 23 years in the overall study population. The 291 infant cord blood samples were collected from families/facilities from day of birth through 6 days of age (median = 0 days). Nine percent of infants were born prematurely with a range of recorded gestational age between 30 and 54 weeks (based on maternal report of date of last menstrual period). Infants included in the serologic substudy study were more likely to be higher SES, term gestation, normal birthweight, and born in non-winter months (March – September) compared to infants in the general study population. Further, infants with sera tested were more likely to be born in a hospital/clinic setting than excluded infants (72% versus 60%).

**Fig. 1:**
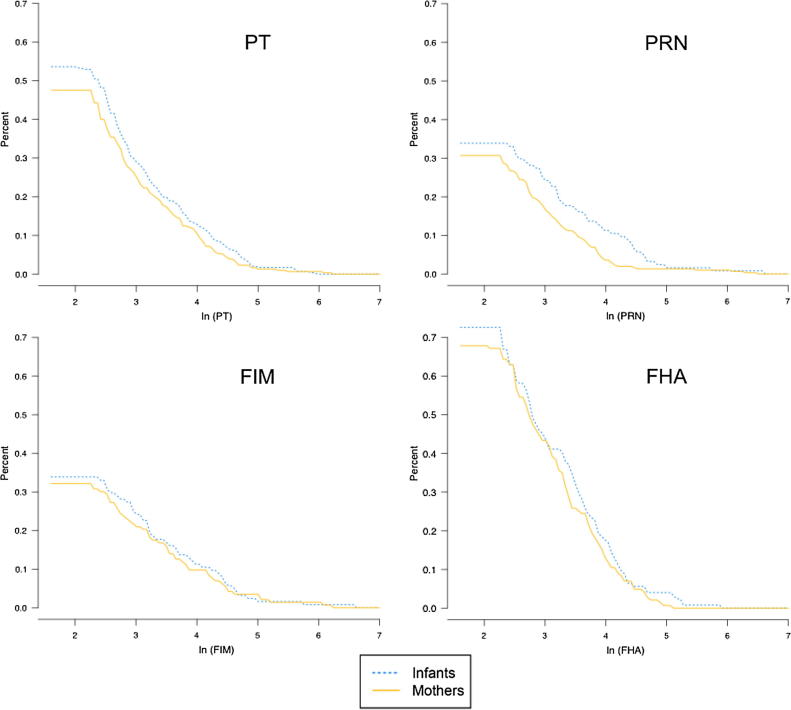
Reverse cumulative distribution curves of four pertussis antibodies for mothers and infants.

Infants had qualitatively higher antibody GMC than mothers for antibody to all pertussis antigens tested, except PRN (Table 1 and Fig. 1). Antibody to FHA had the highest absolute GMC (16.2, 18.2 EU/mL) in mothers and infants, respectively. In our sample, 3.9% (n = 12) of mothers had PT IgG ≥94 EU/mL, indicative of recent pertussis infection.

**Table 1:** Infant and mother antibody levels and transfer efficiency.

						Range	
	n	GMC	GMC Transfer Ratio	95% CI	% Below LOQ	Min	Max
*PT*							
Infant	291	12.7		11.2–14.4	46%	5	377
Mother	305	11.1		9.9–12.5	52%	5	506
Transfer ratio	291		114%	107–120%		17%	2080%

*FIM*							
Infant	124	10.0		8.2–12.3	66%	5	732
Mother	143	9.4		7.9–11.3	68%	5	508
Transfer ratio	120		105%	96–115%		8%	1380%

*FHA*							
Infant	124	18.2		15.1–22.0	27%	5	359
Mother	143	16.2		13.7–19.1	32%	5	161
Transfer ratio	120		110%	101–120%		5%	660%

*PRN*							
Infant	289	7.9		7.1–8.7	71%	5	1588
Mother	303	8.3		7.5–9.2	69%	5	663
Transfer ratio	289		96%	91–100%		10%	380%

Mothers who were not literate and/or of Madhesi ethnicity independently had significantly higher PT and FHA antibody GMC compared to mothers who were literate or of Pahadi ethnicity (Table 2). In contrast, mothers who were literate had significantly higher FIM antibody GMC compared to non-literate mothers. Mothers who gave birth to male versus female infants had higher PRN antibody GMC.

**Table 2:** Comparison of maternal pertussis antibody geometric mean concentration (GMC) by infant, maternal and household characteristics.

	PT				PRN				FHA						FIM	
	n = 305				n = 303				n = 143						n = 143	
	n	(%)	GMC	p-value^a^^,^^b^	n	(%)	GMC	p-value^a^^,^^b^	n	(%)	GMC	p-value^a^^,^^b^	n	(%)	GMC	p-value^a^^,^^b^
Infant Sex				0.96				**0.03**				0.81				0.16
Male	149	49%	10.9		148	49%	9.2		66	46%	15.8		66	46%	10.6	
Female	156	51%	11.3		155	51%	7.6		77	54%	16.5		77	54%	8.5	
Literacy				**0.00**				0.21				**0.01**				**0.05**
Literate	185	65%	9.8		183	65%	8.0		84	64%	13.6		84	64%	11.0	
Non-literate	99	35%	15.0		99	35%	9.0		47	36%	23.1		47	36%	7.4	
Parity				0.92				0.55				0.35				0.73
Nulliparous	124	41%	11.0		124	41%	8.7		58	41%	15.0		58	41%	9.8	
Parous	180	59%	11.2		178	59%	8.0		84	59%	17.3		84	59%	9.3	
Ethnicity				**0.00**				0.25				**0.04**				0.09
Pahadi	180	62%	9.5		178	62%	7.9		83	61%	14.1		83	61%	10.5	
Madhesi	111	38%	14.9		111	38%	9.0		53	39%	20.6		53	39%	7.7	
Socioeconomic status^c^				0.20				0.64				0.13				0.47
Lowest Quartile (Q)	62	21%	15.2		62	21%	8.3		29	21%	22.0		29	21%	8.9	
Bottom/middle Q	67	23%	10.8		66	23%	8.1		33	24%	19.2		33	24%	7.5	
Upper/middle Q	88	30%	10.7		88	30%	9.0		43	32%	14.2		43	32%	10.2	
Highest Q	74	25%	9.7		73	25%	7.7		31	23%	12.8		31	23%	10.9	
Household crowding				0.86				0.39				0.78				0.12
Crowded (>4 people)	129	45%	11.3		128	45%	8.7		61	46%	16.1		61	46%	11.0	
Uncrowded (≤4 people)	159	55%	11.3		158	55%	8.1		73	54%	16.7		73	54%	8.3	

a Wilcoxon rank sum test with continuity correction.

b Bolded values indicate statistical significance (p-value < .05).

c Kruskal-Wallis rank sum test.

**Fig. 2:**
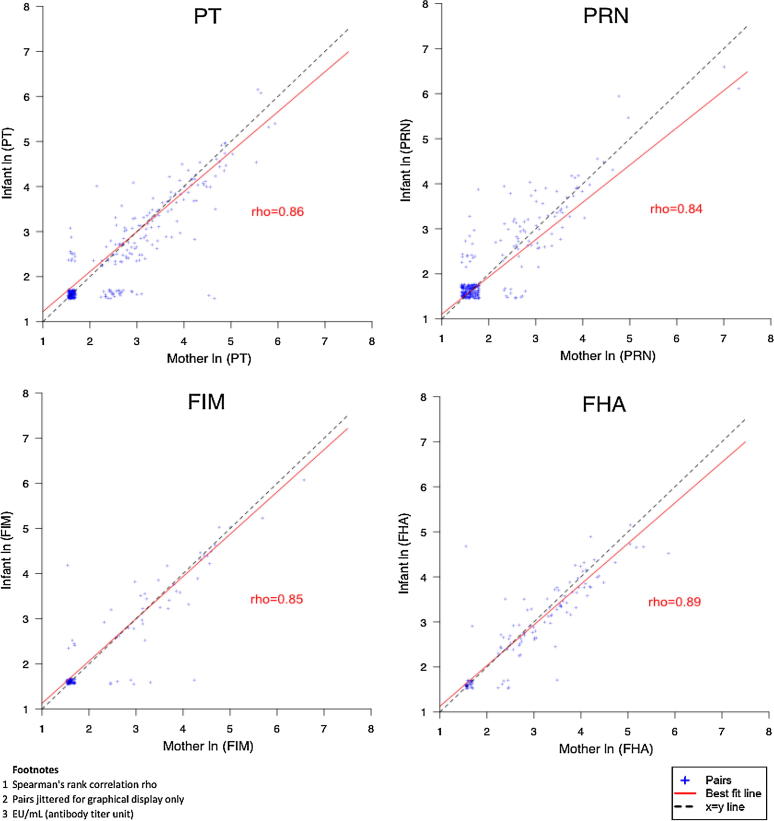
Association of mother and infant pertussis antibodies at birth.

The placental transfer ratio was similar across antibody types ranging from 114% (95% CI: 107–120%) for PT antibody to 96% (95% CI: 91–100%) for PRN antibody (Table 1). Pairs with less than 100% transfer were observed for all 4 antibodies varying from 23% of FHA antibody pairs to 14% of FIM antibody pairs (data not shown). Maternal and infant antibody levels were correlated for all 4 antibodies with the highest correlation for FHA (Spearman’s rank correlation, 0.89) (Fig. 2).

In a multivariable regression model, older gestational age was the only statistically significant variable associated with higher PT antibody transfer (Table 3). For each week of increasing gestational age, the ratio of infant to maternal antibody had an absolute increase of 3% (95% CI: 0–5%). In contrast, maternal to infant transport of FIM antibody decreased by 7% (95% CI: 3–11%) for each additional week of gestation. However this finding was not statistically significant with the removal of one outlier. When analysis was restricted to only pairs where at least one individual had an antibody level above the LOQ, no statistically significant differences in transfer by infant, maternal or household factors were seen (Supplemental Table 1). While the average transfer ratio decreased as maternal antibody levels increased (Supplemental Fig. 2), differences were not statistically significant for mothers with antibody levels at or above the LOQ (data not shown).

**Table 3:** Linear regression for transplacental transfer ratio (natural log transformed).^a^

	PT						PRN						FHA						FIM					
Pair characteristic	No/Mean	(%)/(sd)	TR	e^β^	95% CI	p-value^b^	No/Mean	(%)/(sd)	TR	e^β^	95% CI	p-value^b^	No/Mean	(%)/(sd)	TR	e^β^	95% CI	p-value^b^	No/Mean	(%)/(sd)	TR	e^β^	95% CI	p-value^b^
Sex																								
Male	142	49%	1.17	1 [reference]			141	49%	0.98	1 [reference]			56	47%	1.14	1 [reference]			56	47%	1.12	1 [reference]		
Female	149	51%	1.11	0.94	0.83–1.06	0.29	148	51%	0.94	0.94	0.86–1.04	0.21	64	53%	1.07	0.94	0.77–1.13	0.49	64	53%	0.99	0.83	0.70–0.98	**0.03**
Gestational Age (weeks)	39.4	2.3		1.03^c^	1.00–1.05	**0.05**	39.4	2.3		1.02	1.00–1.04	0.09	39.4	2.0		0.98	0.93–1.03	0.49	39.4	2.0		0.93	0.89–0.97	**0.00**
Birthweight																								
Normal birthweight	233	81%	1.16				232	81%	0.95				97	82%	1.10				97	82%	1.06			
Low birthweight (<2500 g)	54	19%	1.02				53	19%	1.01				21	18%	1.10				21	18%	1.03			
INTERGROWTH-21st SGA																								
AGA	185	68%	1.11				184	68%	0.93				75	65%	1.14				75	65%	1.03			
SGA < 10%	89	32%	1.10				88	32%	0.98				41	35%	1.03				41	35%	1.03			
Literacy																								
Non-literate	94	35%	1.05	1 [reference]			94	35%	0.88	1 [reference]			39	36%	1.11	1 [reference]			39	36%	1.06	1 [reference]		
Literate	177	65%	1.18	1.12^d^	0.97–1.29	0.11	175	65%	1.00	1.12	1.00–1.25	**0.04**	70	64%	1.10	1.17	0.93–1.49	0.18	70	64%	1.09	1.08	0.88–1.33	0.44
Parity																								
Nulliparous	114	39%	1.14	1 [reference]			114	40%	0.96	1 [reference]			44	37%	0.99	1 [reference]			44	37%	1.05	1 [reference]		
Parous	176	61%	1.14	1.00	0.88–1.13	0.96	174	60%	0.96	1.01	0.91–1.11	0.85	75	63%	1.17	1.20	0.98–1.47	0.08	75	63%	1.05	0.99	0.83–1.19	0.95
Ethnicity																								
Madhesi	107	39%	1.09	1 [reference]			107	39%	0.91	1 [reference]			45	40%	1.22	1 [reference]			45	40%	1.04	1 [reference]		
Pahadi	170	61%	1.15	1.00	0.87–1.14	0.99	168	61%	1.00	1.04	0.94–1.16	0.44	68	60%	1.04	0.83	0.66–1.03	0.09	68	60%	1.10	1.08	0.89–1.32	0.43
Household number	5.0	3.2		1.01	1.00–1.03	0.15	4.9	3.5		1.00	0.98–1.01	0.47	5.0	3.6		0.98	0.96–1.01	0.23	5.0	3.6		0.98	0.95–1.00	0.04
Socioeconomic Status																								
Lowest quartile (Q)	61	22%	1.16				61	22%	0.98				26	23%	1.10				26	23%	1.11			
Bottom/middle Q	63	23%	1.16				62	23%	0.95				27	24%	1.20				27	24%	1.08			
Upper/middle Q	84	30%	1.15				84	31%	0.96				34	30%	1.07				34	30%	1.04			
Highest Q	69	25%	1.05				68	25%	0.97				26	23%	1.09				26	23%	1.08			

a Transfer Ratio (TR) = Natural log of infant/mother antibody ratio; full model inclusive of these variables: sex, gestational age, literacy status, parity status, ethnicity, and household number.

b Bolded values indicate statistical significance (p-value < .05).

c Interpretation: For every week increase in gestational age infant to maternal antibody ratio is increased by 3%.

d Interpretation: Infant to maternal antibody ratio is increased 12% for pairs where the mother is literate versus pairs where the mother is non-literate.

## 4 Discussion

Transplacental antibody transfer to four pertussis antigens was efficient with the highest ratio of infant to maternal titers of 1.14. However, a sizeable minority (14–23%) of infants had lower pertussis antibody levels than their mothers. Studies from the 1940 s in unvaccinated mothers found low efficiency of maternal to infant PT IgG transport with only 2–12% of newborns having higher antibody levels than their mothers; however, these results were not generated by ELISA methods and not directly comparable to our results [36]. Recent studies of pertussis antibodies in unvaccinated mothers in various locations, including the US, Asia, and Europe, found efficient antibody transfer between mothers and infants with the following percentages of antibody seen in the infants when compared with the mothers: PT, 107–290%; FHA, 135–183%; PRN, 120–173%; and FIM, 112–157% (ranges exclude preterm infants, when reported separately) [15], [16], [17], [18], [20], [21], [27], [37], [38]. While we also found high correlation between mothers and infants, the transfer in our study population was on the lower end of previously reported values, possibly due to the inclusion of preterm infants. Transplacental transfer studies rarely publish the proportion of pairs with antibody levels in infants below those of their mothers, so we are unable to compare whether the 14–23% of pairs with this transfer is comparable to other unvaccinated populations. Recent data from a population of women vaccinated during pregnancy in Switzerland indicate that 98% of infants were seropositive after 2nd trimester vaccination and 86% after 3rd trimester vaccination [39]. Additional studies are needed in low resource populations to fully characterize the efficiency of transplacental antibody transport.

The factors associated with increased antibody transport in our multivariate models varied by the pertussis antigen. Older gestational age was associated with higher PT antibody transport, consistent with previous results in various populations [17], [20], [40]. A biological rationale for lower transport in preterm infants is that IgG transfer increases during gestation, leaving infants born earlier with less opportunity for maternal transfer [16], [20], [36], [41]. However, recent data in both term and preterm infants suggest that pertussis antibody transfer starts early in the second trimester of pregnancy and increases with gestational age [42].

The inconsistent role of pregnancy history, maternal literacy, and number of household members on transplacental antibody transport has been seen in other studies [18], [19], [20], [22], [27], [39], [40], [43]. Lower health status and infection with HIV or malaria, which are very low in our population, are factors also associated with lower transplacental IgG transport in other studies [18], [40]. The majority of mothers had antibody levels below the LOQ except for FHA antibody, which is known to cross react with other bacterial antigens [35]. A recent US study prior to maternal Tdap administration found comparable levels to ours, with only a fifth of women at delivery having PT antibody levels >5 EU/mL [22]. Approximately 4% of mothers in our study had high PT antibody levels suggesting a recent pertussis infection, a similar prevalence to that reported in Turkey [20]. While there is no serological correlate of immunity, PT IgG antibody titers >94 EU/mL are considered indicative of recent infection [35], [44]. A sizeable proportion of infants had pertussis antibody levels below our LOQ, similar to reports from the USA prior to recommendations for the maternal immunization [22], indicating that majority of infants in Nepal would be susceptible to pertussis.

Our study has several limitations. One is that we were unable to complete testing for all antibodies on all specimens due to limitations on the sample quantity in some subjects. Another limitation is that our study was a convenience sample and we collected blood more frequently for some groups. These groups include infants that were term, normal birthweight, and high SES, which may have potentially biased our study towards higher transfer. However, our findings of no significant differences in transfer by the key variables of gestation, birthweight or SES support minimal bias induced by our study methods. We also do not have childhood pertussis vaccination records for women in our study. However, antibody levels following pertussis vaccination do not persist until adulthood [45]. Finally, we may have had misclassification in our gestational age estimates as they were based on recall of last menstrual period (gestational age of 54 weeks is not biologically plausible). However, we would anticipate that this method would introduce bias towards higher gestational ages and provides a reasonable estimate compared to ultrasound measurement in low-resource settings [46].

Given the low concentration of maternal pertussis antibodies detected in our study, vaccination of pregnant women would be highly likely to increase maternal antibody levels and this antibody would be transplacentally transferred to provide protection in young infants. We note that there is no well-documented antibody level that correlates with protection from pertussis infection or disease [44], [47]. However, it is interesting to speculate that the use of whole cell pertussis vaccine in this setting may be beneficial to the observed low incidence of infant pertussis. Although studies of the epidemiology of pertussis disease among countries or different settings are difficult to compare due to vast differences in testing algorithms and laboratory methods, pertussis outbreaks have been reported nearly exclusively from countries where acellular pertussis vaccine have been utilized. The impact of the type of pertussis vaccine on carriage and transmission of *B. pertussis* is under investigation using primate models [48], with evidence accumulating that acellular pertussis vaccines do not decrease transmission Since the burden of severe disease and deaths from pertussis in infants currently appears to be low in this population [49], maintaining vaccination with whole cell vaccine seems to be warranted and maternal immunization with pertussis vaccine does not appear to be an important medical need in Nepal at this time [50].

## 5 Conclusion

Low pertussis antibody levels were found in mothers and their newborn infants in Sarlahi District, Nepal. The most efficient antibody transferred was PT. Despite the high correlation between mother and infant antibody levels to all four pertussis antigens tested, approximately a fifth of infants had lower antibody levels than their mothers. There was no consistent factor associated with higher maternal antibody levels or more efficient antibody transport from mothers to infants. If the estimated burden of pertussis disease in infancy in Nepal were to increase, maternal immunization should be considered.
