# Supplementary material for: Pertussis seroepidemiology in women and their infants in Sarlahi District, Nepal
Source: Vaccine. 2017 Dec 4;35(48Part B):6766–73. doi: 10.1016/j.vaccine.2017.09.074 (PMC5714611; doi:10.1016/j.vaccine.2017.09.074)
Supplement: Supplementary data 1 [file mmc1.docx]

Flow Diagram description: A total of 664 maternal post-partum samples and 511 cord blood samples were collected between March 1, 2012 and October 30, 2013. Altogether, 315 maternal and 162 infant samples were excluded due to being unpaired. From this initial cohort (N= 349 maternal, 349 infant), an additional 44 maternal samples and 58 infant samples were not tested due to insufficient sera resulting in 305 available maternal samples and 291 available infant samples. Only 291 of the maternal samples had a matched infant samples available resulting in 291 paired mothers and infants. All 291 of these pairs were tested for PT (n=291) and the majority were tested for PRN (n=289), which were the two prioritized assays. Only 120 pairs were tested for FIM and FHA.
