# Supplementary material for: Pertussis seroepidemiology in women and their infants in Sarlahi District, Nepal
Source: Vaccine. 2017 Dec 4;35(48Part B):6766–73. doi: 10.1016/j.vaccine.2017.09.074 (PMC5714611; doi:10.1016/j.vaccine.2017.09.074)
Supplement: Supplementary data 2 [file mmc2.docx]

Supplemental Figure 2: Association of Maternal Antibody Levels with Transfer Ratios
