# Supplementary material for: Pertussis seroepidemiology in women and their infants in Sarlahi District, Nepal
Source: Vaccine. 2017 Dec 4;35(48Part B):6766–73. doi: 10.1016/j.vaccine.2017.09.074 (PMC5714611; doi:10.1016/j.vaccine.2017.09.074)
Supplement: Supplementary file 3 [file mmc3.docx]

Supplemental Table 1. Linear Regression for Ln(Infant:Mother Antibody) - Restricted to those pairs with detectable levels

	PT (n=167)				PRN (n=99)				FHA (n=90)				FIM (n=47)			
**Pair Characteristic**	**No**	**%**	**TR**	**95% CI**	**No**	**%**	**TR**	**95% CI**	**No**	**%**	**TR**	**95% CI**	**No**	**%**	**TR**	**95% CI**
**Overall**	167	100%	1.25	1.13 - 1.4	99	100%	0.88	0.77 - 1.01	90	100%	1.14	1.01 - 1.3	47	100%	1.13	0.89 - 1.43
**Sex**																
Male	81	49%	1.31	1.15 - 1.50	59	60%	0.94	0.80 - 1.11	42	47%	1.20	1.03 - 1.39	27	49%	1.27	0.88 - 1.84
Female	86	51%	1.20	1.03 - 1.39	40	40%	0.79	0.62 - 1.02	48	53%	1.09	0.91 - 1.31	20	51%	0.96	0.73 - 1.25
**Preterm**																
Term	156	93%	1.26	1.14 - 1.40	98	96%	0.88	0.77 - 1.02	82	91%	1.13	1.00 - 1.29	44	93%	1.06	0.85 - 1.33
Preterm (<37 weeks)	11	7%	1.10	0.69 - 1.75	4	4%	0.82	0.23 - 2.88	8	9%	1.17	0.87 - 1.56	3	7%	2.57	0.07 - 95.4
**Birthweight**																
Normal birthweight	133	81%	1.29	1.16 - 1.4	79	82%	0.85	0.73 - 0.99	73	81%	1.14	1.00 - 1.3	40	81%	1.15	0.88 - 1.50
Low birthweight (<2500 grams)	31	19%	1.04	0.83 - 1.3	17	18%	1.04	0.72 - 1.49	17	19%	1.13	0.87 - 1.5	6	19%	1.10	0.64 - 1.90
**INTERGROWTH-21st SGA**																
AGA	100	66%	1.22	1.08 - 1.4	61	67%	0.79	0.67 - 0.94	57	65%	1.19	1.05 - 1.3	31	66%	1.08	0.82 - 1.42
SGA <10%	52	34%	1.18	0.97 - 1.4	30	33%	0.96	0.73 - 1.25	31	35%	1.04	0.79 - 1.3	13	34%	1.11	0.73 - 1.68
**Literacy**																
Non-literate	63	40%	1.08	0.91 - 1.3	36	38%	0.72	0.58 - 0.91	33	40%	1.13	0.87 - 1.5	13	40%	1.20	0.64 - 2.25
Literate	95	60%	1.37	1.20 - 1.5	58	62%	1.01	0.86 - 1.20	49	60%	1.15	1.01 - 1.3	32	60%	1.20	0.99 - 1.46
**Parity**																
Nulliparous	67	40%	1.25	1.06 - 1.47	40	41%	0.89	0.70 - 1.13	27	30%	0.98	0.76 - 1.27	17	40%	1.12	0.85 - 1.48
Parous	100	60%	1.25	1.10 - 1.42	58	59%	0.88	0.74 - 1.05	63	70%	1.21	1.06 - 1.38	30	60%	1.13	0.80 - 1.59
**Ethnicity**																
Madhesi	70	43%	1.15	0.96 - 1.36	38	40%	0.77	0.63 - 0.96	37	44%	1.27	1.10 - 1.48	15	43%	1.14	0.67 - 1.95
Pahadi	91	57%	1.30	1.15 - 1.47	57	60%	1.00	0.83 - 1.19	48	56%	1.06	0.88 - 1.28	30	57%	1.23	1.00 - 1.52
**Household Crowding**																
Crowded (>4 people)	71	44%	1.25	1.05 - 1.50	45	48%	0.83	0.67 - 1.02	37	45%	1.02	0.84 - 1.24	22	44%	1.03	0.80 - 1.33
Uncrowded (≤4 people)	89	56%	1.22	1.08 - 1.37	49	52%	0.96	0.80 - 1.15	46	55%	1.25	1.06 - 1.47	23	56%	1.39	0.98 - 1.97
**SES**																
Lowest quartile (Q)	42	26%	1.24	1.04 - 1.48	23	24%	0.96	0.71 - 1.30	21	25%	1.12	0.76 - 1.64	12	26%	1.25	0.65 - 2.39
Bottom/middle Q	40	25%	1.27	1.03 - 1.56	18	19%	0.84	0.60 - 1.17	23	27%	1.23	1.01 - 1.51	7	25%	1.36	0.88 - 2.10
Upper/middle Q	44	27%	1.30	1.03 - 1.65	33	35%	0.90	0.70 - 1.14	23	27%	1.10	0.86 - 1.40	12	27%	1.12	0.72 - 1.74
Top Q	35	22%	1.10	0.91 - 1.34	21	22%	0.91	0.67 - 1.23	18	21%	1.14	0.98 - 1.32	14	22%	1.16	0.85 - 1.58
